# Drinking water source and human *Toxoplasma gondii* infection in the United States: a cross-sectional analysis of NHANES data

**DOI:** 10.1186/1471-2458-14-711

**Published:** 2014-07-10

**Authors:** Whitney S Krueger, Elizabeth D Hilborn, Reagan R Converse, Timothy J Wade

**Affiliations:** 1 Oak Ridge Institute for Science and Education, Oak Ridge, Tennessee, USA; 2 Environmental Public Health Division, Office of Research and Development, US Environmental Protection Agency, 104 Mason Farm Road, Chapel Hill, North Carolina, USA

**Keywords:** Nutrition surveys, Toxoplasma, Drinking water, Water wells, Cross-sectional studies

## Abstract

**Background:**

*Toxoplasma gondii* imparts a considerable burden to public health. Human toxoplasmosis can be life-threatening in immunocompromised individuals, has been associated with psychiatric disorders, and can cause severe congenital pathologies, spontaneous abortion, or stillbirth. Environmental modes of transmission contributing to the incidence of human toxoplasmosis are poorly understood. We sought to examine National Health and Nutrition Examination Survey (NHANES) data for risk factors associated with *T. gondii* seroprevalence.

**Methods:**

*T. gondii* serology results reported for Continuous NHANES survey years 1999–2004 and 2009–10 were examined. To explore associations with toxoplasmosis seropositivity, covariates of interest were selected *a priori*, including source and home treatment of tap water. Associations between potential risk factors and evidence of IgG antibodies against *T. gondii* were assessed using multivariable logistic regression.

**Results:**

Among 23,030 participants with available *T. gondii* serology across 8 years of continuous NHANES survey data (1999–2004; 2009–2010), persons born outside the United States were significantly more likely to be seropositive, and seropositivity was inversely associated with years spent in the United States. Among US-born participants, participants with homes on well water (both those who used at-home water treatment devices and those who did not), as well as participants with public/private company-provided tap water who did not use at-home water treatment devices, were significantly more likely to be seropositive compared to participants who used home treatment devices on tap water provided by a private or public water company. A comparative subpopulation analysis revealed age-adjusted seroprevalence among US-born persons 12-49 yrs old significantly declined to 6.6% (95% CI, 5.2-8.0) (*P* <0.0001) in 2009–10, compared to previously published reports for NHANES data from 1988–1994 (14.1%) and 1999–2004 (9.0%).

**Conclusions:**

Data suggests that *T. gondii* infections continue to decline in the United States, but the overall infection rate remains substantial at nearly 7%. Despite the limitations in the Continuous NHANES cross-sectional survey, the association between well water use and *T. gondii* infection warrants further research.

## Background

*Toxoplasma gondii,* an obligate intracellular protozoan, is one of the most widespread zoonotic parasites in the world. *T. gondii* infects all warm-blooded animals, but utilizes domestic and wild cats in the Felidae family as its definitive host [1-3]. *T. gondii* infects humans via ingestion of undercooked meat containing trophozoites or tissue cysts, or through ingestion of sporulated oocysts found in food, soil, and water recently contaminated with cat feces [1,4,5]. When oocysts are ingested by an intermediate host such as humans [6], they migrate in the host’s body and form permanent cysts in various organs and tissues, but most commonly in the brain [7]. Toxoplasmosis is a lifelong infection that has the potential to reactivate and cause severe disease and even death in immunocompromised individuals.

Though primary toxoplasmosis is usually subclinical, sequelae can range from mild flu-like symptoms to seizures and confusion. Severity of symptoms may depend upon the parasite stage ingested, with infections from oocysts believed to elicit more clinically severe manifestations than ingestion of tissue cysts [4]. Immunocompromised individuals can experience more severe symptoms, either during primary infection or following reactivation of latent cysts [8,9]. Likely attributed to cysts in the brain, psychiatric disorders including mood disorders, personality changes, and schizophrenia have been associated with chronic *T. gondii* infections; however, the underlying etiology is not entirely clear [10,11]. Of special public health concern, fetuses can be infected via vertical transmission when a woman experiences a primary infection during pregnancy. Disseminating tachyzoites colonize placental tissues and infect the fetus, potentially causing severe injury, stillbirth, or spontaneous abortion. Congenital toxoplasmosis results in the highest burden of disease for human *T. gondii* infections [6].

*T. gondii* has infected an estimated 25-30% of the world’s population [9]; however prevalence varies across countries/geographical regions [12]. North America has a lower prevalence (10-30%) compared to Latin America and tropical regions of Africa, which have a 50-80% prevalence. In addition to felid presence and density, climatic, anthropogenic, economic, social, and cultural factors (e.g. hygienic practices, dietary habits, cooking methods), as well as water quality and sanitation coverage all play important roles in *T. gondii* environmental persistence, zoonotic transmission, and prevalence in humans [1]. In the United States, toxoplasmosis is responsible for one of the highest direct costs to health care among diseases partially associated with water-borne transmission [13].

Because recent epidemiological studies could not attribute the majority of *T. gondii* infections to contaminated food consumption or soil-related exposures [14,15], other sources of contamination are likely starting to play a key role in transmitting *T. gondii* to humans, especially in the United States [16]*.* In the environment, oocysts can resist freezing and moderately high temperatures to remain viable for years in soil and water [4,17]. Water chemical and physical treatments, including chlorination and ozone treatment, are not effective in killing oocysts; however, oocysts are relatively susceptible to ultraviolet radiation [18,19]. Although modern municipal water treatment systems (with filtering, coagulation, flocculation, and settling) are generally effective at removing oocysts [4], many populations do not have access to drinking water that has undergone this level of treatment. Drinking water is an endemic source of *T. gondii* in Brazil and Guatemala, and drinking water has been implicated as the source of toxoplasmosis outbreaks in several countries [4], including Panama [20] and Canada [21,22]. Oocysts have not been directly detected in US drinking water, but assessing environmental contamination of *T. gondii* is technically difficult [23]. Researchers have hypothesized that ingesting inadequately treated drinking water may be a significant source for human infections [4,24].

The National Health and Nutrition Examination Survey (NHANES), within the National Center for Health Statistics of the Centers for Disease Control and Prevention, has conducted large scale surveys designed to assess changes over time of the health and nutritional status of adults and children in the United States. Descriptive epidemiology reports have previously reported *T. gondii* seroprevalence trends for past NHANES cohorts (1988–1994 and 1999–2004) [25-28]. Increased odds of seropositivity were reported with increasing age, poverty, foreign country of birth, lower level of education, living in crowded conditions, and working in soil-related occupations, which varied by race/ethnicity [25]. To assess the disease burden in the United States, a comparative subpopulation analysis of US-born adults aged 12–49 years revealed that *T. gondii* seroprevalence declined from 14.1% during the Third NHANES (1988–1994) to 9.0% during the Continuous NHANES cycles from 1999–2004 [27].

Because the CDC recently released seroprevalence data for *T. gondii* among eligible participants in the Continuous NHANES 2009–10 cycle, we examined all available Continuous NHANES data (1999–2004 and 2009–10) to expand on the previous literature and look for evidence of environmental risk factors associated with *T. gondii* seroprevalence among a nationally representative population [3].

## Methods

Four 2-year cycles of Continuous NHANES data (1999–2000, 2001–02, 2003–04, and 2009–10) were examined [3]. Available serum from eligible participants 6-49 yrs old (1999–2004) and ≥6 yrs old (2009–2010) were tested by the CDC for immunoglobulin G (IgG) antibodies against *Toxoplasma gondii* by an enzyme immunoassay (EIA). A dichotomous seropositivity cut-off was provided by the CDC (Continuous NHANES 1999–2004: IgG ≥10 IU/mL = positive; IgG <10 IU/mL = negative; Continuous NHANES 2009–2010: IgG ≥33 IU/mL = positive; IgG <33 IU/mL = negative).

Data were collected using a complex survey design; therefore, seroprevalence estimates were weighted to represent the total US civilian non-institutionalized population, and CDC’s recommended approaches for SAS survey analysis procedures were followed [3]. All analyses were conducted using SAS v9.3 (Cary, NC). Using all of the available NHANES survey data, the authors *a priori* selected potential covariates of interest to examine associations with toxoplasmosis seropositivity. Of special interest were blood biomarkers (using complete blood count results) to examine clinical severity and exposure to drinking water, because these factors had not yet been thoroughly examined in previous reports. Smoking status was based on serum cotinine level (ng/mL) to delineate light smokers and second-hand exposure (10-99 ng/mL) from smokers (≥100 ng/mL). The NHANES variable of ‘household food security’ was determined using the US Food Security Survey Module. Affirmative responses were counted from 18 items for households with children and 10 items for households without children. Categorical data were derived: no affirmative responses = full food security; 1–2 = marginal food security; 3–5 (without children) or 3–7 (with children) = low food security; 6–10 (without children) and 8–18 (with children) = very low food security. Items included: worried will run out of food, food didn’t last, couldn’t afford meals, adults cut size or skip meals, eat less than should, hungry but didn’t eat, adults don’t eat whole day, and use of emergency foods from food banks, soup kitchens, or other organizations. For our analyses, we combined all categories below ‘full food security’ to create a dichotomous variable.

In the Housing Characteristics (HOQ) questionnaire participants were asked: *“What is the source of tap water in this home? Is it a private or public water company, a private or public well, or something else?”* and *“Are any of the water treatment devices listed on this card used in your home?”* Treatment devices listed were: Brita® or other pitcher water filter, ceramic or charcoal filter, water softener, aerator, and reverse osmosis. In order to better understand their interaction, the two variables associated with tap water were combined into one covariate with 4 categorical responses (home-treated well water, home-untreated well water, home-treated water company and home-untreated water company).

Seroprevalence of IgG antibodies against *T. gondii* was assessed with multivariable logistic regression to examine associations with key demographic characteristics and potential risk factors associated with seropositivity. Wald chi-square odds ratio (OR) estimates and 95% confidence intervals (CI) were ascertained for unadjusted and adjusted regression models. Covariates with p values <0.1 were considered for inclusion in multivariable models. Multivariable models were selected using stepwise backwards elimination of covariates with p values > 0.1. Selected models included all covariates with p values < 0.05 as well as any *a priori* selected demographic characteristics that confounded observed associations between independent variables and the outcome.

## Results

Across the 8 years of Continuous NHANES data for which *T. gondii* seroprevalence was examined (1999–2000, 2001–02, 2003–04, and 2009–10), *T. gondii* serology results were available for 23,030 participants (Table 1). Approximately 5,300 participants 6-49 yrs old were included in each of the first 3 Continuous NHANES cycles (1999–2000, 2001–02, and 2003–04). For the more recent cycle (2009–10), the eligible population was expanded to all participants ≥6 yrs old; therefore, this cycle had a larger subgroup of over 7,000 participants. *T. gondii* seroprevalence was also significantly different across key demographic variables, including age, gender, race/ethnicity, and poverty index (Table 1). Controlling for age, there was a statistically significant difference in overall *T. gondii* seroprevalence among the survey years, (*P* <0.0001) with adjusted odds of seropositivity in both 2001–02 and 2009–10 cycles significantly lower compared to the 1999–2000 cycle (data not shown).

**Table 1:** ***Toxoplasma gondii *** **seroprevalence**^**a **^**by demographic characteristics for participants ≥6 years old; NHANES 1999–2004, 2009-10**^**b**^

**Variable**	**N**	**Prevalence (%)**^**c**^	**95% CL**	***P***
Overall	23030	2642 (11.7)	10.8,12.6	
Continuous NHANES survey year				0.0116
1999-2000	5089	602 (13.3)	11.6,15.1	
2001-2002	5751	527 (9.3)	7.1,11.4	
2003-2004	5120	434 (10.8)	8.6,13.0	
2009-2010	7070	1079 (13.2)	11.9,14.4	
Age group (years)				<0.0001
6-12	4274	155 (3.5)	2.5,4.4	
13-17	4693	279 (4.6)	3.7,5.6	
18-28	4810	488 (9.2)	7.7,10.7	
29-43	4799	757 (14.0)	12.4,15.6	
44-80	4450	963 (17.7)	16.0,19.4	
Gender				0.0133
Male	11239	1402 (12.6)	11.4,13.8	
Female	11791	1240 (10.8)	9.8,11.9	
Race/ethnicity				<0.0001
Non-Hispanic White	8546	854 (9.9)	8.8,11.0	
Non-Hispanic Black	5535	566 (12.6)	10.7,14.5	
Mexican American	6441	749 (13.6)	11.7,15.5	
Other Hispanic	1490	366 (24.8)	19.7,30.0	
Other, incl multi-racial	1018	107 (12.9)	10.1,15.7	
Poverty index^d,e^				<0.0001
Below	5807	744 (14.3)	12.5,16.1	
At or above	15454	1621 (10.9)	9.9,11.8	
Country of origin^d^				<0.0001
United States	18358	1401 (8.9)	7.9,9.9	
Mexico	2661	614 (24.1)	21.4,26.7	
Elsewhere	2005	627 (29.2)	25.7,32.7	

^a^For Continuous NHANES 1999–2004: IgG ≥10 IU = positive; IgG <10 IU = negative. For Continuous NHANES 2009–2010: IgG ≥33 IU/mL = positive; IgG <33 IU/mL = negative. ^b^*T. gondii* serology performed only among participants ages 6–49 for 1999–2004 data. ^c^All prevalence estimates were weighted. ^d^Covariate has some missing data. ^e^Family income-to-poverty ratio (FIPR) categorized as 0.00–0.99 = below poverty; 1.00 and above = at or above poverty.

Risk factor associations were first assessed for all 23,030 participants using multivariable logistic regression (Table 2). Increasing age was significantly associated with *T. gondii* seropositivity, and men were more likely to be seropositive than women (adjusted OR [AOR] = 1.2; 95% CI, 1.1-1.4). Compared to non-Hispanic Whites, Mexican Americans were less likely to be seropositive (AOR = 0.5; 95% CI, 0.4-0.7), while non-Hispanic Blacks and other Hispanics had significantly elevated adjusted odds of seroprevalence (AOR = 1.3; 95% CI, 1.04-1.6 and AOR = 1.6; 95% CI, 1.3-2.1; respectively). Compared to full food security, having marginally secure or insecure household food was associated with higher odds of seropositivity (AOR = 1.3; 95% CI, 1.1-1.5). Smokers had higher odds of seropositivity compared to non-smokers (AOR = 1.2; 95% CI, 1.03-1.5). In addition, the largest effect was observed for country of birth origin. Compared to participants born in the United States, participants born in Mexico had significantly higher odds of seropositivity (AOR = 6.3; 95% CI, 4.7-8.4), as did participants born in a foreign country other than Mexico (AOR = 3.7; 95% CI, 3.0-4.6). In examining health correlates, an inverse association was observed with participants’ self-reported general health condition – the worse the perceived health condition, the higher odds of seropositivity (Table 2). Participants who reported taking treatment for anemia in the past 3 months were more likely to have antibodies against *T. gondii* (AOR = 1.4; 95% CI, 1.01-2.0). In unadjusted comparisons there was a significant association between seropositivity and eosinophilia, but the effect diminished in the multivariable model.

**Table 2:** **Risk factors associated with antibodies**^**a **^**against *Toxoplasma gondii *** **among participants ≥6 years old; NHANES 1999–2004, 2009-10**^**b**^

**Variable**	**N**	**IgG positive**^**c**^	**Unadjusted OR**^**d**^	**Adjusted OR**^**d**^
		**n (%)**	**(95% CI)**	**(95% CI)**
Survey year				
1999-2000	5089	602 (13.3)	Ref	Ref
2001-2002	5751	527 (9.3)	**0.7 (0.5-0.9)**^**e**^	**0.7 (0.5-0.9)**
2003-2004	5120	434 (10.8)	0.8 (0.6-1.0)	0.8 (0.6-1.1)
2009-2010	7070	1079 (13.2)	1.0 (0.8-1.2)	**0.6 (0.5-0.7)**
Age				
*Continuous (years)*	23030	2642 (11.7)	**1.03 (1.03-1.03)**	**1.03 (1.03-1.04)**
Age group (years)				
6-12	4274	155 (3.5)	Ref	--
13-17	4693	279 (4.6)	**1.4 (1.03-1.8)**	--
18-28	4810	488 (9.2)	**2.8 (2.3-3.5)**	--
29-43	4799	757 (14.0)	**4.5 (3.6-5.8)**	--
≥44	4450	963 (17.7)	**6.0 (4.5-8.0)**	--
Gender				
Female	11791	1240 (10.8)	Ref	Ref
Male	11239	1402 (12.6)	**1.2 (1.04-1.4)**	**1.2 (1.1-1.4)**
Race/Ethnicity				
Non-Hispanic White	8546	854 (9.9)	Ref	Ref
Non-Hispanic Black	5535	566 (12.6)	**1.3 (1.1-1.6)**	**1.3 (1.04-1.6)**
Mexican American	6441	749 (13.6)	**1.4 (1.2-1.7)**	**0.5 (0.4-0.7)**
Other Hispanic	1490	366 (24.8)	**3.0 (2.3-4.0)**	**1.6 (1.3-2.1)**
Other, incl. multiracial	1018	107 (12.9)	**1.3 (1.02-1.8)**	0.7 (0.5-1.0)
Household food security^f^				
Full food security	15104	1593 (10.9)	Ref	Ref
Marginally secure or insecure	7212	973 (14.5)	**1.4 (1.2-1.6)**	**1.3 (1.1-1.5)**
Smoking status^f,g^				
Non-smoker	18438	1977 (11.0)	Ref	Ref
Light smoker/moderate second-hand exposure	1387	194 (13.0)	1.2 (1.0-1.5)	1.1 (0.9-1.4)
Smoker	3045	447 (14.1)	**1.3 (1.1-1.6)**	**1.2 (1.03-1.5)**
Country of origin^f^				
United States	18358	1401 (8.9)	Ref	Ref
Mexico	2661	614 (24.1)	**3.2 (2.7-3.9)**	**6.3 (4.7-8.4)**
Elsewhere	2005	627 (29.2)	**4.2 (3.4-5.2)**	**3.7 (3.0-4.6)**
Source and home treatment^h^ of tap water^f^				
Home-treated private/public water company	4129	420 (9.9)	Ref	Ref
Home-untreated private/public water company	16089	1855 (12.1)	1.2 (1.1-1.4)	**1.2 (1.003-1.4)**
Home-treated well water	1056	143 (12.7)	**1.3 (1.05-1.7)**	**1.4 (1.1-1.8)**
Home-untreated well water	1107	143 (12.3)	1.3 (0.9-1.8)	**1.5 (1.02-2.2)**
General health condition^f^				
Excellent	6341	477 (8.5)	Ref	Ref
Very good	6306	588 (9.8)	**1.2 (1.0-1.4)**	**1.1 (0.9-1.3)**
Good	7090	920 (13.3)	**1.7 (1.4-1.9)**	**1.2 (1.04-1.4)**
Fair	2835	552 (19.2)	**2.6 (2.2-2.9)**	**1.5 (1.3-1.8)**
Poor	447	103 (25.1)	**3.6 (2.6-5.0)**	**1.9 (1.4-2.7)**
Took treatment for anemia in past 3 months^f^				
No	22397	2526 (11.5)	Ref	Ref
Yes	625	113 (18.5)	**1.7 (1.3-2.3)**	**1.4 (1.01-2.0)**
Blood eosinophils number^f^				
* Continuous (1000 cells/uL)*	22940	2629 (11.7)	**1.4 (1.02-1.9)**	1.3 (1.0-1.7)

^a^For Continuous NHANES 1999–2004: IgG ≥10 IU = positive; IgG <10 IU = negative. For Continuous NHANES 2009–2010: IgG ≥33 IU/mL = positive; IgG <33 IU/mL = negative. ^b^*T. gondii* serology performed only among participants ages 6–49 for 1999–2004 data. ^c^All prevalence estimates were weighted. ^d^Wald Chi-square logistic regression used. ^e^Bolded font denotes statistically significant data (*P* < 0.05). ^f^Covariate has some missing data. ^g^Based on serum cotinine level (ng/mL); nonsmoker <10, light smoker/second-hand exposure 10–99, smoker ≥100. ^h^Types of home water treatment devices included: Brita® or other pitcher water filter, ceramic or charcoal filter, water softener, aerator, reverse osmosis.

Associations between *T. gondii* seroprevalence, tap water source, and using home water treatment devices were observed (Table 2). Compared to participants using home-treated company water, participants with home-untreated well water had a 1.5 times higher adjusted odds (95% CI, 1.02-2.2) of *T. gondii* seropositivity. Because there was a high correlation between seropositivity and country of origin, we theorized that if seropositive participants born outside the United States were more likely to be infected while living abroad (where risk of *T. gondii* infection is higher), then their source of drinking water once living in the United States would not have the same association with *T. gondii* seropositivity. To test this hypothesis, we next examined the association of drinking water and IgG antibodies against *T. gondii* separately among US-born and foreign-born participants (Table 3).

**Table 3:** **Risk factors associated with antibodies**^**a **^**against *Toxoplasma gondii *** **among participants ≥6 years old by country of birth; NHANES 1999–2004, 2009-10**^**b**^

**Variable**	**US-born**				**Foreign-born**			
	**N**	**IgG positive**^**c**^	**Unadjusted OR**^**d**^	**Adjusted OR**^**d**^	**N**	**IgG positive**^**c**^	**Unadjusted OR**^**d**^	**Adjusted OR**^**d**^
		**n (%)**	**(95% CI)**	**(95% CI)**		**n (%)**	**(95% CI)**	**(95% CI)**
Survey year								
1999-2000	4010	320 (9.7)	Ref	Ref	1078	282 (32.3)	Ref	Ref
2001-2002	4699	276 (7.4)	0.7 (0.5-1.1)	0.7 (0.5-1.1)	1052	251 (21.8)	**0.6 (0.4-0.9)**^**e**^	**0.6 (0.4-0.9)**
2003-2004	4248	231 (7.9)	0.8 (0.5-1.2)	0.8 (0.5-1.2)	872	203 (28.0)	0.8 (0.5-1.3)	0.8 (0.6-1.1)
2009-2010	5401	574 (10.3)	1.1 (0.8-1.4)	**0.6 (0.4-0.7)**	1664	505 (27.6)	0.8 (0.6-1.2)	**0.7 (0.5-0.9)**
Age								
*Continuous (years)*	18358	1401 (8.9)	**1.03 (1.03-1.04)**	**1.04 (1.03-1.04)**	4666	1241 (27.5)	**1.02 (1.01-1.02)**	**1.03 (1.02-1.04)**
Gender								
Female	9481	652 (8.2)	Ref	Ref	2307	588 (26.7)	Ref	Ref
Male	8877	749 (9.6)	**1.2 (1.002-1.4)**	**1.2 (1.05-1.5)**	2359	653 (28.2)	1.1 (0.9-1.3)	1.1 (0.9-1.4)
Race/Ethnicity								
Non-Hispanic White	8131	756 (9.2)	Ref	Ref	415	98 (22.8)	Ref	Ref
Non-Hispanic Black	5104	419 (10.0)	1.1 (0.9-1.4)	1.2 (0.9-1.5)	430	147 (38.3)	**2.1 (1.4-3.1)**	**1.9 (1.3-2.9)**
Mexican American	3773	140 (3.8)	**0.4 (0.3-0.5)**	**0.5 (0.4-0.7)**	2666	609 (23.9)	1.1 (0.8-1.4)	0.9 (0.7-1.2)
Other Hispanic	727	37 (4.3)	**0.4 (0.3-0.8)**	**0.5 (0.3-0.98)**	760	329 (44.1)	**2.7 (2.0-3.6)**	**2.3 (1.7-3.0)**
Other, incl. multiracial	623	49 (10.8)	1.2 (0.8-1.8)	1.5 (1.0-2.3)	395	59 (15.4)	**0.6 (0.4-0.9)**	**0.5 (0.4-0.8)**
Household food security^f^								
Full food security	12523	942 (8.7)	Ref	Ref	2581	651 (25.0)	Ref	Ref
Marginally secure or insecure	5246	414 (9.3)	1.1 (0.9-1.3)	**1.3 (1.01-1.6)**	1962	559 (33.1)	**1.5 (1.2-1.8)**	1.2 (1.0-1.5)
Renting vs. owning home								
Owned/being bought	10861	877 (9.3)	Ref	--	1956	459 (23.7)	Ref	Ref
Rented/other arrangement	7332	498 (8.0)	0.9 (0.7-1.0)	--	2666	769 (30.7)	**1.4 (1.2-1.7)**	**1.3 (1.1-1.6)**
Smoking status^f,g^								
Non-smoker	14427	933 (7.7)	Ref	Ref	4005	1044 (27.3)	Ref	--
Light smoker/2^nd^ hand exposure	1082	105 (9.3)	1.2 (0.9-1.6)	1.2 (0.9-1.6)	305	89 (29.4)	1.1 (0.8-1.6)	--
Smoker	2717	351 (12.9)	**1.8 (1.5-2.1)**	**1.3 (1.1-1.6)**	329	96 (26.7)	1.0 (0.7-1.4)	--
Water source^f^								
Home-treated^h^ water company	3267	194 (6.9)	Ref	Ref	861	226 (26.2)	Ref	Ref
Home-untreated water company	12608	922 (8.7)	**1.3 (1.05-1.6)**	**1.2 (1.01-1.5)**	3476	933 (28.3)	1.1 (0.9-1.4)	1.0 (0.7-1.3)
Home-treated well water	990	131 (12.4)	**1.9 (1.4-2.6)**	**1.6 (1.2-2.1)**	66	12 (19.4)	0.7 (0.3-1.8)	0.5 (0.2-1.0)
Home-untreated well water	1016	119 (12.0)	**1.8 (1.3-2.7)**	**1.7 (1.1-2.4)**	91	24 (19.8)	0.7 (0.4-1.3)	0.8 (0.4-1.4)
General health condition^f^								
Excellent	5456	293 (6.8)	Ref	Ref	885	184 (21.8)	Ref	Ref
Very good	5372	365 (8.0)	1.2 (1.0-1.5)	1.0 (0.8-1.3)	932	223 (24.2)	1.2 (0.9-1.5)	1.1 (0.8-1.5)
Good	5360	450 (9.8)	**1.5 (1.2-1.9)**	1.1 (0.9-1.4)	1728	470 (28.5)	**1.4 (1.1-1.8)**	1.2 (0.9-1.6)
Fair/poor	2164	292 (14.9)	**2.4 (1.9-3.0)**	**1.5 (1.1-1.9)**	1116	363 (36.1)	**2.0 (1.6-2.6)**	**1.6 (1.2-2.2)**
Took treatment for anemia in past 3 mos^f^								
No	17859	1334 (8.7)	Ref	--	4533	1192 (27.3)	Ref	Ref
Yes	493	65 (14.8)	**1.8 (1.3-2.6)**	--	131	48 (40.4)	**1.8 (1.1-3.1)**	**1.8 (1.1-3.0)**
Blood eosinophils number (1000 cells/uL)^f^								
Normal (<0.35)	15960	1239 (8.9)	Ref	--	4061	1040 (26.4)	Ref	Ref
Above normal (≥0.35)	2441	158 (8.6)	1.0 (0.8-1.2)	--	573	192 (35.1)	**1.5 (1.2-1.9)**	**1.5 (1.2-1.8)**
Percentage of lifetime lived in US^f^								
≥0.76	--	--	--	--	1147	206 (18.3)	Ref	Ref
0.61-0.75	--	--	--	--	1044	295 (29.7)	**1.9 (1.4-2.5)**	**1.9 (1.4-2.5)**
0.41-0.60	--	--	--	--	1149	337 (29.5)	**1.9 (1.4-2.4)**	**2.2 (1.6-2.8)**
≤0.40	--	--	--	--	1175	364 (32.5)	**2.2 (1.7-2.7)**	**2.5 (1.9-3.4)**

^a^For Continuous NHANES 1999–2004: IgG ≥10 IU = positive; IgG <10 IU = negative. For Continuous NHANES 2009–2010: IgG ≥33 IU/mL = positive; IgG <33 IU/mL = negative. ^b^*T. gondii* serology performed only among participants ages 6–49 for Continuous NHANES 1999–2004 data. ^c^All prevalence estimates were weighted. ^d^Wald Chi-square logistic regression used. ^e^Bolded font denotes statistically significant data (*P* < 0.05). ^f^Covariate has some missing data. ^g^Based on serum cotinine level (ng/mL); nonsmoker <10, light smoker/second-hand exposure 10–99, smoker ≥100. ^h^Types of home water treatment devices included: Brita® or other pitcher water filter, ceramic or charcoal filter, water softener, aerator, reverse osmosis.

### US-born participants

The final multivariable model included NHANES survey year, age, gender, household food security, race/ethnicity, smoking status, general health condition and source/treatment of tap water (Table 3). Mexican Americans and other Hispanics were less likely to be seropositive compared to non-Hispanic Whites (AOR = 0.5; 95% CI, 0.4-0.7 and AOR = 0.5; 95% CI, 0.3-0.98; respectively). Smokers had a higher odds of seropositivity compared to non-smokers (AOR = 1.3; 95% CI, 1.1-1.6), and participants who reported fair or poor health were more likely to be seropositive (AOR = 1.5; 95% CI, 1.1-1.9) than participants who reported very good or good health, when compared to participants who reported excellent health. Participants with homes on well water (both those who used at-home water treatment devices on the well water [AOR = 1.6; 95% CI, 1.2-2.1] and those who did not [AOR = 1.7; 95% CI, 1.1-2.4]), as well as participants using home-untreated company tap water (AOR = 1.2; 95% CI, 1.01.-1.5) had significantly higher odds of seropositivity compared to participants using home-treated tap water provided by a private or public water company.

To expand on the comparative subpopulation analysis previously reported by Jones et al., among US-born persons 12–49 years old, *T. gondii* age-adjusted seroprevalence significantly fell to 6.6% (95% CL, 5.2-8.0) (*P* <0.0001) for the 2009–10 NHANES cycle, compared to 14.1% (95% CL, 12.7-15.5) during the Third NHANES (1988–1994) and 9.0% (95% CL, 7.5-10.4) during the Continuous NHANES 1999–2004 [27] (data not shown).

### Foreign-born participants

After adjusting for residential environment, the final multivariable model included NHANES survey year, age, gender, household food security, race/ethnicity, percentage of lifetime lived in the United States, smoking status, general health condition, source of tap water, anemia treatment, and eosinophilia (Table 3). In contrast to the US-born participants, among foreign-born participants, a higher *T. gondii* seroprevalence was observed among Hispanics other than Mexican American (AOR = 2.3; 95% CI, 1.7-3.0) and non-Hispanic Blacks (AOR = 1.9; 95% CI, 1.3-2.9). A lower seroprevalence was observed among “other” races, including multiracial (AOR = 0.5; 95% CI, 0.4-0.8). When considering the percentage of one’s lifetime spent living in the United States, a clear trend was observed, where the lower the percentage of lifetime, the higher the likelihood of *T. gondii* seropositivity (AOR = 2.5; 95% CI, 1.9-3.4 for living ≤40% of lifetime compared to ≥76%). In regards to health status, as observed in US-born participants, foreign-born participants who reported a fair or poor general health condition were more likely to be seropositive (AOR = 1.6; 95% CI, 1.2-2.2). In addition, participants with a blood eosinophil count above the normal range (≥3500 cells/uL) and participants who reported taking treatment for anemia in the past 3 months were significantly more likely to be seropositive (AOR = 1.5; 95% CI, 1.2-1.8 and AOR = 1.8; 95% CI, 1.1-3.0; respectively). Source of tap water and using home water treatment devices were not associated with *T. gondii* seroprevalence among foreign-born participants.

## Discussion

We observed statistically significant increased odds of *T. gondii* seropositivity among US-born NHANES participants whose tap water source was well water (both home treated and untreated), as well as home-untreated company water, compared to participants who used home-treated water from a private or public water company. The absence of significant associations between tap water source/treatment and *T. gondii* seropositivity among those not born in the United States provides additional evidence that drinking water may be an important source of toxoplasmosis in the United States.

Although *T. gondii* oocysts are resistant to chlorine and other types of disinfection [4], because of their relatively large size (10–12 μm), they should be removed by standard municipal treatment consisting of coagulation, flocculation, and settling prior to filtration [29]. While outbreaks of *T. gondii* have only been attributed to municipal waters using unfiltered water systems (Brazil and Canada), outbreaks could also occur following a water treatment breakdown or operation error [30]. In addition, *T. gondii* oocysts have been found to be widespread in wells located on farms [31] and can survive for long periods in soil and water [24]. A study conducted among farms in Poland reported an association between well water consumption and toxoplasmosis IgG seropositivity [31]. Not only was a positive correlation observed between drinking well water and *T. gondii* seropositivity among persons living on the farms (*P* < 0.05), but *T. gondii* DNA was found significantly more frequently in water samples collected from windlass-operated wells (37.5%) than water from deep wells with a pump (6.2%) and from water supply systems (0%). Well water, especially private or from a small shared source, is probably much less likely to be filtered adequately to remove *T. gondii* oocysts. Although we lacked much necessary detail to draw definitive conclusions, our results are consistent with an association between well water and *T. gondii* infection.

Because the information collected on the type of water treatment was limited, it is difficult to interpret the effect of additional home treatment. Some treatments included in the question such as water softeners, would not have any effect on *T. gondii* oocysts, and even most water filters are simply designed to reduce taste and odor or reduce some chemicals such as lead and chlorine. Because oocysts are 10–12 μm in size [32], some standard Brita® filters, such as faucet (0.5 micron) and pitcher (1 micron) filters are expected to effectively remove oocysts, but other filters are only designed for taste and odor improvement and would have no effect. Moreover, the effectiveness of home treatments is impacted by maintenance and upkeep of the filter system. The use of additional treatment may also signal increased awareness and concern of water contamination and such participants may be more careful about general hygiene and the water they choose to drink.

Our analyses confirmed previous reports that those born outside the United States had higher odds for *T. gondii* seropositivity, supporting the evidence that other countries have a higher endemicity of human toxoplasmosis. We further demonstrated that the percentage of one’s lifetime lived in the United States was inversely associated with *T. gondii* infection. Due to the NHANES classification of country of birth origin, we were unable to conduct meaningful analyses of which countries represent a higher risk of *T. gondii* infection. Seroprevalence varied among different race/ethnicity groups when stratified by country of origin, which is consistent with previous reports [25,28]. Stratification by country of origin also suggested blood eosinophils above the normal range and reported treatment for anemia in the past 3 months were associated with *T. gondii* seropositivity for foreign-born participants, but not for US-born participants. While these factors may simply be correlates of infection and not clinically associated with *T. gondii* infections, molecular and experimental infection studies have discovered that *T. gondii* genotypes vary in virulence, and these genetic lineages have evolved in different regions of the world [1]. Highly clonal genotypes II and III, the primary lineages in North America and Europe are considered to be avirulent in immunocompetent persons. In contrast, atypical genotypes resulting from frequent genetic exchanges and genetic drift have been associated with greater clinical severity. High genetic diversity among *T. gondii* isolates has been found in South America, Africa, and Asia. Because the clinical symptoms of anemia and inflammation were observed only among seropositive foreign-born NHANES participants, perhaps those born outside the United States acquired toxoplasmosis from more virulent strains. Following ingestion, *T. gondii* parasites invade the gastrointestinal wall, disseminate, and elicit a strong immune response that triggers significant ancillary pathologies; anemia and eosinophilia have been reported in clinically severe cases of toxoplasmosis [33-37].

While the Continuous NHANES collects a wealth of information for a national, representative sample of the United States population, we do recognize its limitations. The cross-sectional survey design prevents us from inferring causality. In addition, combining datasets across multiple survey years can be a limitation, depending on the covariates of interest. Questions and medical exams are not constant across the survey years: response options change, questions/medical tests are added, and questions/medical tests are dropped. In addition, several questionnaires and medical exams are administered to only subgroups (e.g. adults ≥20 years old). Because previous papers of *T. gondii* seroprevalence among NHANES participants analyzed subgroups that had additional NHANES data, e.g. education level and occupation among adults ≥20 years old [26], our goal was to maintain the largest cohort as possible for consistency and increased power.

Unfortunately, there was very limited data for other risk factors of interest, including the presence of *T. gondii* in drinking water sources, occupations with soil and water exposure, mental health status (e.g. depression and schizophrenia), and overall dietary behaviors (e.g. eating undercooked meat). In addition, as a health and nutrition survey, NHANES does not collect data regarding exposure to recreational waters, a key interest in environmental *T. gondii* transmission. Antibodies against *T. gondii* have been found in several species of marine mammals off of both US coastlines [23], suggesting that freshwater runoff is contaminating sea water [38]. This raises the question about the risk to humans from accidentally swallowing contaminated water during recreational exposure to rivers, lakes, and ocean waters [4]. Despite these limitations, this study provides risk factor data that can inform future environmental assessments and prospective human studies to better link cause and effect for water-associated toxoplasmosis.

## Conclusions

Our findings are consistent with national trends observed in previous studies of *T. gondii* seroprevalence among NHANES participants. Seroprevalence is declining in the United States, with only 6.6% of the US-born population 12–49 years of age having serological evidence of *T. gondii* infection during 2009–10, compared to 9.0% during 1999–2004 and 14.1% during 1988–1994. We similarly observed differences in seropositivity by country of birth origin and race/ethnicity. There was also a significant association with less than excellent self-reported general health condition and seropositivity. Furthermore, this is the first paper to report associations between tap water source and *T. gondii* among US-born residents; although, a casual association cannot be demonstrated due to the cross-sectional design of NHANES. In addition, because standard *T. gondii* serological assays cannot differentiate environmentally-acquired oocyst infections from foodborne tissue cyst infections, recently developed oocyst antigen-specific assays, including the enzyme-linked immunoassay developed by Hill et al. [39], are valuable tools to more accurately assess the disease burden of environmental exposures to *T. gondii*. In addition, further investigations are warranted to examine how well water may be contaminated with oocysts, what role well systems may play in maintaining and spreading oocysts, and which home water treatment devices are effective in eliminating oocysts from tap water.

## Competing interests

The authors declare that they have no competing interests.

## Authors’ contributions

All authors have substantially contributed to this work. WSK, RRC, and TJW performed the data processing and statistical analyses. All authors wrote, edited, and approved the final manuscript.

## Authors’ information

The views expressed in this paper are those of the authors and do not necessarily reflect the views or policies of the U.S. Environmental Protection Agency. Mention of trade names or commercial products does not constitute endorsement or recommendation for use.

## Pre-publication history

The pre-publication history for this paper can be accessed here:

http://www.biomedcentral.com/1471-2458/14/711/prepub
